# Helium ventilation is safe and feasible in ICU patients admitted after cardiac arrest

**DOI:** 10.1186/cc10886

**Published:** 2012-03-20

**Authors:** D Brevoord, C Beurskens, N Juffermans, W Van den Bergh, W Lagrand, B Preckel, J Horn

**Affiliations:** 1 Academic Medical Centre, University of Amsterdam, the Netherlands

## Introduction

Most patients admitted to the ICU after cardiac arrest die or have an unfavourable neurological outcome due to brain damage. Currently, the only treatment to reduce brain injury after cardiac arrest is mild hypothermia. Helium inhalation has shown promising results as a neuroprotective agent in animal models of cerebral infarction. If helium inhalation ameliorates neurological damage by reducing reperfusion injury in humans as well, this could be of great benefit to patients. As no studies exist that investigate the use of helium ventilation in patients after cardiac arrest we investigated whether this treatment is safe and feasible.

## Methods

A single-centre open-label intervention study was performed in a mixed 30-bed academic ICU, approved by the local medical ethics committee. Inclusion criteria: admission after a witnessed cardiac arrest, presenting with ventricular fibrillation or tachycardia, return of spontaneous circulation within 30 minutes, treatment with hypothermia. Exclusion criteria: pre-existing neurological disorders or the need for a FiO_2 _>50% or >10 mmHg PEEP on ICU admission. Helium was administered during 3 hours as a 1:1 mixture with oxygen, using a Servo-i ventilator. An independent data safety monitoring board reviewed all problems arising from the helium ventilation itself and all fatalities. Poor outcome was assessed with the Glasgow Outcome Score at 30 days: death and vegetative state were defined as poor outcome. Data are presented as mean ± SD or numbers and proportions.

## Results

In total 25 patients were included, 20 (80%) male, age 64.8 ± 12.1 years, APACHE II score 20.0 ± 8.6, SAPS II 53.6 ± 18.6. Helium treatment was started 4:57 ± 0:54 hours after arrest. In one patient the treatment was stopped due to inadequate ventilation using the preset limits. This was not due to the helium ventilation and no adverse events due to helium ventilation were noted. Overall, nine (36%) patients had a poor outcome.

## Conclusion

In this small study, we encountered no problems associated with helium treatment in patients admitted to the ICU after cardiac arrest. This opens the way for studies investigating the hypothesis that helium treatment reduces neurological injury in these patients.

